# How predation shapes the social interaction rules of shoaling fish

**DOI:** 10.1098/rspb.2017.1126

**Published:** 2017-08-30

**Authors:** James E. Herbert-Read, Emil Rosén, Alex Szorkovszky, Christos C. Ioannou, Björn Rogell, Andrea Perna, Indar W. Ramnarine, Alexander Kotrschal, Niclas Kolm, Jens Krause, David J. T. Sumpter

**Affiliations:** 1 Department of Zoology, Stockholm University, Stockholm, Sweden; 2 Department of Mathematics, Uppsala University, Uppsala, Sweden; 3 School of Biological Sciences, University of Bristol, Bristol, UK; 4 Department of Life Sciences, Roehampton University, London, UK; 5 Department of Life Sciences, The University of the West Indies, St Augustine, Trinidad and Tobago; 6 Faculty of Life Sciences, Albrecht Daniel Thaer-Institut, Humboldt-University zu Berlin, Invalidenstrasse 42, 10115 Berlin, Germany; 7 Leibniz-Institute of Freshwater Ecology and Inland Fisheries, Department of Biology and Ecology of Fishes, Müggelseedamm 310, 12587 Berlin, Germany

**Keywords:** group living, collective motion, *Poecilia reticulata*, collective behaviour, interaction rules

## Abstract

Predation is thought to shape the macroscopic properties of animal groups, making moving groups more cohesive and coordinated. Precisely how predation has shaped individuals' fine-scale social interactions in natural populations, however, is unknown. Using high-resolution tracking data of shoaling fish (*Poecilia reticulata*) from populations differing in natural predation pressure, we show how predation adapts individuals' social interaction rules. Fish originating from high predation environments formed larger, more cohesive, but not more polarized groups than fish from low predation environments. Using a new approach to detect the discrete points in time when individuals decide to update their movements based on the available social cues, we determine how these collective properties emerge from individuals' microscopic social interactions. We first confirm predictions that predation shapes the attraction–repulsion dynamic of these fish, reducing the critical distance at which neighbours move apart, or come back together. While we find strong evidence that fish align with their near neighbours, we do not find that predation shapes the strength or likelihood of these alignment tendencies. We also find that predation sharpens individuals' acceleration and deceleration responses, implying key perceptual and energetic differences associated with how individuals move in different predation regimes. Our results reveal how predation can shape the social interactions of individuals in groups, ultimately driving differences in groups' collective behaviour.

## 1. Introduction

Predation is often considered to be the major selective force driving the origin and maintenance of group living [1–3]. Both theoretical and empirical studies demonstrate that an individual's *per capita* risk is lower in larger and more cohesive groups, reducing individual risk through dilution [4–6], attack abatement [7,8], and confusion effects [9]. Evidence that predation drives the formation of larger and more cohesive groups has come from a number of comparative studies between populations or groups exposed to varying degrees of predation pressure [10–16]. Cohesive and coordinated group behaviours emerge, however, from the decision rules that individuals use to interact in groups, and how predation has shaped these fine-scale social decisions is still unclear.

In many moving animal groups, these social decisions are characterized by simple interaction rules, such as attraction and alignment with near neighbours, that allow individuals to remain cohesive and coordinated while on the move together [17–19]. It has previously been demonstrated that predators can select for cohesive and coordinated moving groups, when predatory fish preferentially targeted simulated prey that had lower degrees of social attraction and alignment with near neighbours [20]. Theoretical studies also show that predation can lead to different interaction rules being selected for, subsequently creating distinct macroscopic properties of groups [21]. But exactly how predation has shaped the social interaction rules within animal groups in the wild is still unknown. Now, using highly quantitative movement data from real animal groups [22–25], we can decode how individuals are interacting within them. Further, by comparing the social interaction rules of animals that have been subject to varying degrees of predation over their evolutionary and life histories, we may now determine in detail how natural predation shapes individuals' social interactions.

The Trinidadian guppy (*Poecilia reticulata*) is a classic evolutionary study system often used to investigate how predation has shaped the life-history and behavioural traits of individuals [26]. Using simple aggregation measures, Seghers [13] and subsequently others, have demonstrated that fish living in high predation environments form more cohesive shoals than fish living in low predation environments [12,13]. Using high resolution trajectory data on the movements of fish originating from both high and low predation environments, here we quantify how predation has shaped the social decisions that produce these differences. We first ask whether the likelihood of individuals leaving or joining groups differs between fish from high and low predation populations. We go on to quantify differences in the shape, structure, and directional organization of fish shoals from the high or low predation populations. We then ask how these macroscopic properties emerge from differences in individuals' social interaction rules. Previous methods for inferring interaction rules in animal groups have applied an averaging procedure, where the movements of animals between successive recorded points in an animal's trajectory have been interpreted as discrete movement decisions. While these methods have been informative, they do not differentiate between the long uninformative portions of trajectories when animals continue on their course without interacting with neighbours, and the few discrete times when animals update their position based on the available social cues [27,28]. To link our understanding of collective motion to perceptual and cognitive processes, therefore, we require new analytical techniques to decipher exactly when and how individuals in moving groups decide to update their position [29,30]. In this study, we use a new method to detect when individuals *decide* to update their position based on the available social cues, and then ask how these decisions have been shaped by natural predation.

## 2. Material and methods

### (a) Experimental methods

Wild adult guppies from four rivers (Aripo, Turure, Quare, and Tunapuna/Tacarigua—tributaries of the same river) were collected from the Northern mountain range, Trinidad, in March 2015. Within each river, we collected fish from a high predation site and a low predation site. High predation sites contain either the main predator of adult guppies, *Crenicichla frenata*, or other predatory fish species (*Hoplias malabaricus* or *Aequidens pulcher*). Low predation sites did not contain these species, but contained *Rivulus hartii* which is not considered to be a major predator of adult guppies [26]. The dispersal of predatory fishes within the rivers appears to have been limited by natural barriers, such as waterfalls, occurring along the rivers [26]. Therefore, high predation sites and low predation sites are found respectively further downstream or upstream along the rivers. As well as differing in predation regimes, these high and low predation sites can also differ in environmental factors such as canopy cover, water depth, and the spectral properties of the water. However, there is consensus that these differences are either less important, or augment the effects of predation in driving life-history and behavioural differences between fish from these populations [31–33].

Fish were transported back to aquaria facilities at the University of West Indies and were housed in glass tanks at 24°C and fed flake food ad libitum at the start and middle of each day to maintain satiation levels. Fish were held for at least 36 h before experimentation. Trials were run between 08.00 and 17.30 each day. Groups of either two or eight fish of the same sex, representing group sizes naturally found in the wild [34], were selected and placed into a holding tube in the corner of a visually isolated rectangular arena (1 000 × 900 mm). The arena was filled with aged water to a depth of 45 mm and 1 l of water from the housing tanks was added to ensure that conspecific chemical cues remained relatively consistent between trials. After the fish had been in the holding tube for 5 min, we remotely lifted the holding tube allowing the fish to explore the arena. The fish were allowed to explore the arena for ∼16 min. Trials were filmed at 24 frames per second at a resolution of 1 920 × 1 080 pixels using a Nikon D700 camera placed directly above the arena. We determined the size of each fish by taking photographs of the fish in each trial, subsequently measuring them using a bespoke script in MATLAB. No fish were re-used between trials. In total, we recorded ∼73 h of footage of shoals of two fish (*n* = 115 male trials, *n* = 109 female trials) and ∼35 h of footage of shoals of eight fish (*n* = 51 male trials, *n* = 78 female trials).

### (b) Analysis

We tracked the pairs of fish using CTrax [35] semi-automated tracking software and corrected any errors the software made using the Fixerrors GUI in MATLAB. We tracked the groups of eight fish using Didson Tracking Software [36] in MATLAB. From the trajectories of the groups of eight fish, we measured a number of group level properties that characterized the structure and broad-scale social dynamics of the shoals. From the trajectories of the pairs, we analysed how individuals were interacting with their partner while moving together. All analyses were done using bespoke scripts in MATLAB (2016). For full details of analyses, see the electronic supplementary material.

### (c) Statistics

We modelled all response variables using generalized linear mixed effects models. These were fitted with predation regime (high or low), sex, subgroup size (where applicable), and body size (see electronic supplementary material, figure S1) as fixed effects. Because males and females and fish from high or low predation environments differ in body size (see electronic supplementary material, figure S1), we wanted to ensure that differences in body size would not drive any interpretation of the differences in behaviour of fish between high and low predation environments. Therefore, to control for this, we included the body size of fish as a covariate in all statistical models. River (nested within predation and crossed with sex) and trial (where applicable) were fitted as random factors to the data. Sex, predation, and subgroup size (where applicable) were treated as categorical variables in all analyses, whereas body size was treated as continuous. We performed all analyses in R. Full details of all statistical models, analyses, and tables can be found in the electronic supplementary material.

## 3. Results

### (a) Group level properties

**Figure 1.**
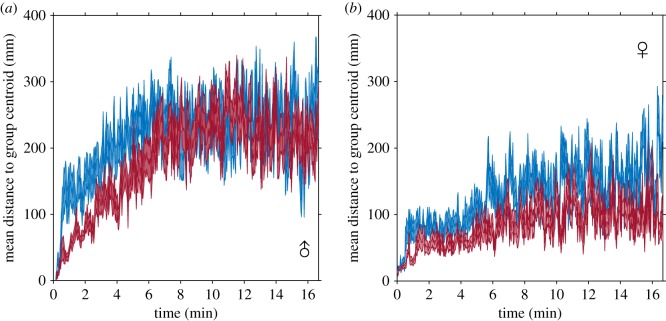
Mean (±1 s.e.) distance individuals were from the group's centroid for shoals of eight (*a*) male or (*b*) female fish. Shoals from high predation environments are shown in red and shoals from low predation environments are shown in blue.

**Figure 2.**
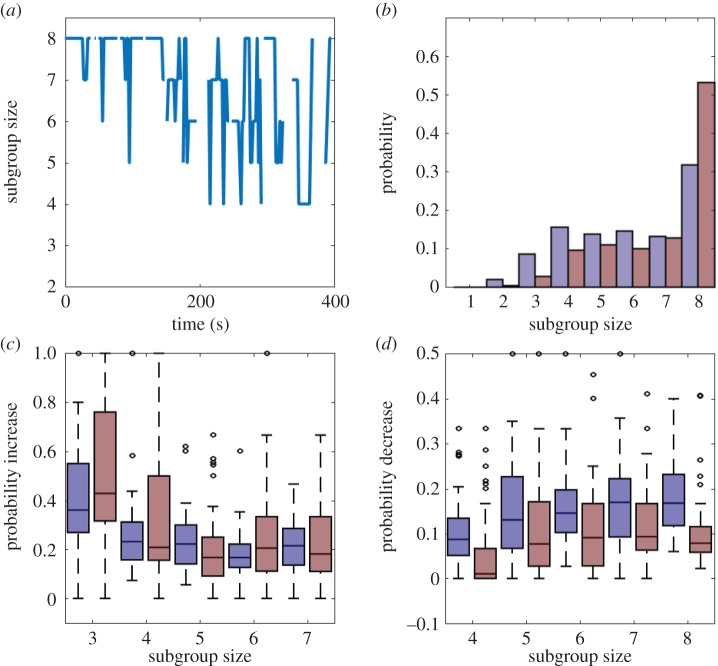
(*a*) Example of the number of fish in the main subgroup for the first 400 s of one of the trials with eight female fish. Fish generally break up into smaller subgroups over the course of the trial. (*b*). Probability distribution of the largest subgroup size for fish from low (blue) or high (red) predation populations. (*c*, *d*) Probability that fish from low (blue) or high (red) predation populations join (*c*) or leave (*d*) the largest subgroup while exploring the arena. Fish from high predation populations are less likely to depart the group, whereas the joining probabilities between populations is the same (electronic supplementary material, table S2). The horizontal lines in the centre of each box denotes the median of each category, while the bottom and top edges of each box denote the 25th and 75th percentiles, respectively. Whiskers extend to the data points that are not considered outliers (black circles). Subgroups in this figure were classified as fish that were within 100 mm of at least one neighbour (see the electronic supplementary material).

Before analysing the fine-scale interactions of pairs of fish, we first quantified the broad-scale social dynamics of groups of eight fish, and asked whether the structure of these groups differed between fish from high or low predation populations. Fish from high predation populations formed more cohesive groups than fish from low predation populations, especially during the early stages of the trials (figure 1*a*,*b*). As the trials progressed, the distance to the centre of the group centroid increased in both males and females from high and low predation populations (figure 1). The increase in distance to the group centroid over time was due to the fish breaking off into smaller subgroups. These subgroups merged and split (figure 2*a*), similar to the fission–fusion behaviour guppies exhibit in the wild [34]. Guppies from high predation populations were more likely to be found together in a group of eight fish than guppies from low predation populations (*p*_MCMC_ = 0.012; figure 2*b*; electronic supplementary material, table S1).

To investigate the decisions driving the distributions of subgroup sizes, we determined the size of the largest subgroup that was exploring the arena, and assessed how this subgroup changed in size over discretized time points (2 s). While the probability of individuals joining the largest subgroup was not different between predation regimes (*p*_MCMC_ = 0.59; figure 2*c*; electronic supplementary material, table S2), the probability that group members would depart the largest subgroup was lower for fish from high predation populations (*p*_MCMC_ = 0.026; figure 2*d*; electronic supplementary material, table S2).

**Figure 3.**
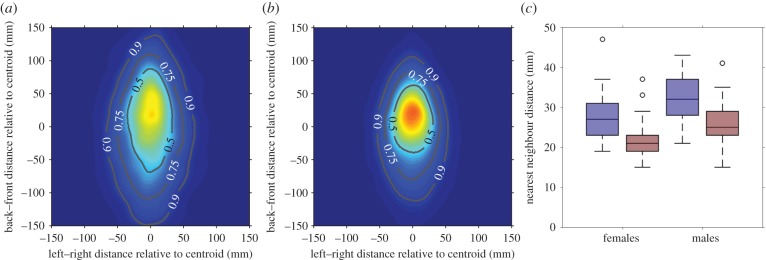
Shape of shoals of eight female fish from low predation (*a*) or high predation (*b*) populations. Contour lines represent regions containing the proportion of total observations where individuals were found relative to the shoal centroid located at (0, 0). Shoals from high predation populations were generally more compact than shoals from low predation populations. These patterns were consistent in shoals of eight male fish (electronic supplementary material, figure S2) and across different subgroup sizes (electronic supplementary material, figure S3). (*c*) Modal nearest neighbour distances were also smaller for fish from high predation environments (red) compared with low predation environments (blue). This was consistent across females (left) and males (right). See electronic supplementary material, figure S4 for a breakdown of modal nearest neighbour distances by river. The horizontal lines in the centre of each box denotes the median of each category, while the bottom and top edges of each box denote the 25th and 75th percentiles, respectively. Whisker extends to the data points that are not considered outliers (black circles).

While these leaving and joining decisions describe the broad-scale social dynamics of guppy shoals, they do not examine how a group is structured when individuals are together. Guppies formed elliptical shoals with the length of the shoal generally being larger than its width (females: figure 3*a*,*b* and males: electronic supplementary material, figure S2*a*,*b*). Both the width and length of shoals from high predation populations were smaller than the width and length of shoals from low predation populations (width: *χ*^2^ = 4.9, d.f. = 1, *p* = 0.03; length: *χ*^2^ = 10.5, d.f. = 1, *p* = 0.001; electronic supplementary material, figure S3). Fish from high predation populations similarly had smaller modal nearest neighbour distances than fish from low predation populations (figure 3*c*; electronic supplementary material, figure S4; *χ*^2^ = 14.6, d.f. = 1, *p* < 0.001).

Predation is not only expected to shape how cohesive groups are, but also coordination between group members. In particular, predation is expected to make individuals in groups align with their near neighbours, as these alignment responses may allow information transmission [37] or increase the confusion effect [20,38,39]. To investigate this, we measured a group's polarization as a function of its speed. While groups travelling faster were more polarized (electronic supplementary material, figure S5), we found no difference in the polarization of groups between high or low predation populations (females: *χ*^2^ = 0.34, d.f. = 1, *p* = 0.56; males: *χ*^2^ = 1.09, d.f. = 1, *p* = 0.30). Further, we found no evidence that fish from high predation populations spent more time in a highly polarized state (polarization scores above 0.85; *p*_MCMC_ = 1.0), or moved more quickly than fish from low predation populations (*χ*^2^ = 0.1, d.f. = 1, *p* = 0.75). Predation, therefore, appears to increase shoal cohesion, but not directional alignment in these fish.

### (b) Individuals' interactions in pairs

The differences observed in group level properties between fish from high or low predation populations are a consequence of the movement decisions that individuals use to interact with their neighbours. To investigate in more detail how fish interact with their neighbours, we studied the movements of same sex pairs in the arena used for the groups of eight fish. We can be sure that in pairs, the interactions between the two fish are a result of each others' movements, and not some function of more than one neighbour. As with the groups of eight fish, pairs of fish from high predation were closer together than fish from low predation populations (*χ*^2^ = 9.89, d.f. = 1, *p* = 0.002; electronic supplementary material, figure S6). In addition to a predation effect, smaller fish also had smaller nearest neighbour distances than larger fish (*χ*^2^ = 4.77, d.f. = 1, *p* = 0.03).

**Figure 4.**
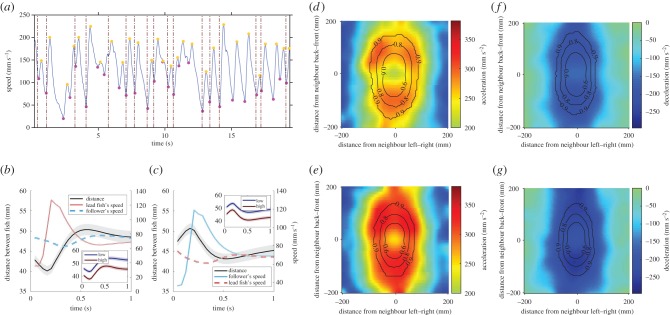
(*a*) Example of a fish's typical speed profile. For each fish's speed profile, we determined the times when it decided to ‘update its position’ by detecting the times when the fish's speed was in a trough (magenta points). We also detected the maximum speeds associated with these decisions (yellow points). Many of these decisions were associated with a change in angle immediately prior to the increase in speed (dashed vertical lines; see electronic supplementary material, figure S7). (*b*) Speed profile of the fish at the front of the pair (red line refer to right *Y*-axis) when it decides to move (at 0 s). The lead fish decides to move when the distance between the fish reaches ∼43 mm (averaged over both high and low predation males), but these distances are significantly lower in fish from high predation populations (red line in insert) than fish from low predation populations (blue line in insert). (*c*) Speed profile of the follower (light blue line refer to right *Y*-axis) when it decides to move. The follower speeds up when the distance between the fish reaches ∼47 mm (averaged over both high and low predation males), but again, these distances are significantly lower in fish from high predation populations (red line in insert) than fish from low predation populations (blue line in insert). Error bars in (*b*,*c*) represent mean standard error per trial (only partially visible for speed due to low variation). Data are only for male fish, but females also show the same movement profile, with similar separation distances (electronic supplementary material, figure S9). (*d*, *e*) Acceleration of male fish from low (*d*) or high (*e*) predation populations as a function of neighbour position. (*f*, *g*) Deceleration of male fish from low (*f*) or high (*g*) predation populations as a function of neighbour position. In each of these plots, the fish making the decision is located at (0,0) and facing along the positive *Y*-axis. Male fish from high populations have higher acceleration and deceleration than their low predation counterparts. Data from lead fish and followers are combined within these plots as they show similar symmetry around *y* = 0. Contour lines represent the proportion of observations of neighbours in those respective positions.

To understand how fish from high predation populations reduce their separation distances, we first aimed to classify how guppies typically shoal, regardless of any predation effects. Guppies swim with a saltatory movement style, with intermittent bursts of speed (figure 4*a*), typical of many species of fish [40]. Many of these speed bursts are accompanied by a change in angle immediately prior to the speed increase (figure 4*a*; electronic supplementary material, figure S7). The discrete nature of these bursts and turns leads us to refer to these changes in speed and angle as movement *decisions*. We identified all the decisions made by each fish, and then asked how and when fish were updating their positions as a function of their neighbour's position and movements. Indeed, other recent methods have begun to use similar approaches to classify the collective motion of fish shoals [41–43].

The distance between the fish on the left–right axis was typically stable at ∼15–20 mm, but varied on the front–back axis (electronic supplementary material, figure S8). The decisions of each fish in the pair to move depended on their relative distance apart. If the fish in front of its partner was less than ∼43 mm ahead, then the lead fish accelerated (figure 4*b*). The lead fish continued to accelerate until it reached a speed of ∼130 mm s^−1^, at which point it decelerated. When the distance between the fish reached ∼46 mm, the follower accelerated (figure 4*c*) with a similar acceleration profile as the lead fish. This simple attraction–repulsion interaction acted to maintain cohesion while pairs moved together asynchronously.

With an understanding of how the guppies adjusted their speed as a function of the neighbour's relative position, we then asked whether these movement decisions differed between fish from high or low predation populations. Sixteen per cent of decisions in males and 13% of decisions in females resulted in the follower ‘overtaking’ the fish in front. While fish from high predation populations performed more overtaking events than fish from low predation populations, this effect was not statistically significant (*p*_MCMC_ = 0.09). The mean distance between the pair when one of the fish decided to move was lower for fish from high predation populations than from low predation populations (*χ*^2^ = 7.13, d.f. = 1, *p* = 0.008; inserts figure 4*b*,*c*). This combination of more overtaking events and reduced initiation distances explains why the high predation pairs were typically closer together.

The distance a fish moved during a decision (i.e. the distance it travelled from the start of one decision to the start of the next decision) did not differ between fish from high or low predation environments (*χ*^2^ = 0.13, d.f. = 1, *p* = 0.72), however, the acceleration during the decision did. Fish from high predation populations had larger acceleration than fish from low predation populations (*χ*^2^ = 5.21, d.f. = 1, *p* = 0.02; figure 4*d*,*e*; electronic supplementary material, figure S9*c*,*d*). Fish from high predation population environments are also known to have larger acceleration than fish from low predation environments during escape responses [44]. Therefore, differences in the acceleration of fish from high or low predation environments might not be socially driven, and instead may simply be a characteristic of how these fish move. To investigate this, we measured the acceleration of the fish when they were at different distances from their partner. If differences in the acceleration between fish from high and low predation populations were socially motivated, then we would not expect to see differences in the acceleration of fish from high and low predation when the fish were further apart. There remained a difference between the acceleration of high and low predation males when they were separated by more than 200 mm (*χ*^2^ = 8.0, d.f. = 1, *p* = 0.005; electronic supplementary material, figure S10*b*). While there was no difference in the acceleration of females from high and low predation environments when fish were separated by more than 200 mm (*χ*^2^ = 0.1, d.f. = 1, *p* = 0.76; electronic supplementary material, figure S10*a*), females were rarely separated by more than 200 mm. At least in males, therefore, the higher acceleration of fish from high predation environments seem to be typical of how the fish swim, regardless of social effects.

Guppies often use their pectoral fins during forward motion [45,46], and we sometimes observed the fish using active braking; deceleration caused by flaring of the pectoral fins. This is indicative that at least some of their movements also involve decisions to stop moving. In females, the average deceleration of a fish was related to their body size, but not predation regime (body size: *χ*^2^ = 10.1, d.f. = 1, *p* = 0.002; predation: *χ*^2^ = 0.34, d.f. = 1, *p* = 0.56; electronic supplementary material, figure S9*e*,*f*). In males, however, fish from high predation populations had larger deceleration than fish from low predation populations (*χ*^2^ = 9.7, d.f. = 1, *p* = 0.002; figure 4*f* ,*g*). To investigate whether these differences in deceleration between high and low predation males were socially driven, again we investigated the deceleration of fish as a function of the distance from their partner. There was also a difference between the deceleration of males from high and low predation environments when fish were separated by more than 200 mm (*χ*^2^ = 6.61, d.f. = 1, *p* = 0.01; electronic supplementary material, figure S12B). Like these fish's acceleration, therefore, larger deceleration in the males from high predation environments do not appear to be socially driven.

Despite fish from high predation environments having larger acceleration and deceleration than fish from low predation environments, high predation fish were not less synchronized than low predation fish in the timing of their decisions. We measured the time lag between when one fish made a decision to the time when its partner made a decision. There was no difference in these response times between high or low predation males (*χ*^2^ = 1.9, *p* = 0.17) or females (*χ*^2^ = 0.27, *p* = 0.60). We also measured whether there was a difference in the number of decisions individuals made per second between fish from high and low predation populations. While it appeared that males from high predation populations made more decisions per second, this could be explained on the basis that smaller males made more decision per second than larger males (*χ*^2^ = 4.45, d.f. = 1, *p* = 0.035). On the other hand, females from high predation populations made fewer decisions per second that females from low predation populations (*χ*^2^ = 7.92, d.f. = 1, *p* = 0.005) with no effect of body size on this decision rate (*χ*^2^ = 0.38, d.f. = 1, *p* = 0.54).

**Figure 5.**
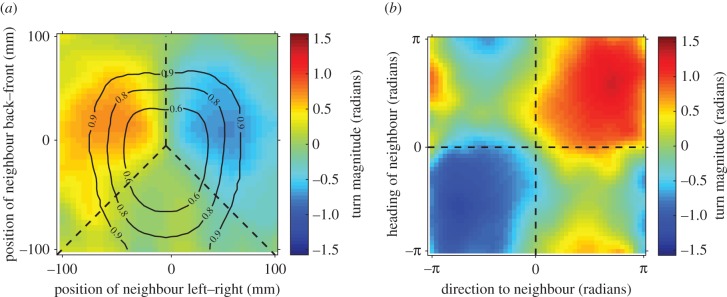
(*a*) Mean turning angle of a fish as a function of its neighbour's position, averaged across both sexes and predation regimes as all categories showed similar responses. The fish making the turning decision is located at (0, 0) and facing along the positive *Y*-axis. Fish turn left when their neighbour is ∼45° and on their left, turn right when their neighbour is ∼45° and on their right. They have approximately equal proportions of left and right turns when their neighbour is behind them. Contour lines represent the proportion of observations of neighbours in those respective positions. (*b*) Turning response of a focal fish as a function of its neighbour's direction (*X*-axis) or heading (*Y*-axis). Data in this figure are averaged across males and females and across populations, as all fish showed similar turning profiles. The dashed lines in both panels separate regions of interest that were analysed in statistical models.

Forty-one per cent of the decisions to speed up were accompanied by the fish turning. These changes in direction occurred immediately before a fish decided to increase its speed (figure 4*a*). All fish, regardless of predation regime or sex showed similar turning responses to their partner's position (figure 5*a*). Fish most often turned left when their partner was on the left, most often turned right if their partner was on the right, with equal turns to the left and right if their partner was behind them (figure 5*a*). The turning responses of guppies, therefore, can be broken down into three 120° regions as a function of partner position, as denoted by the dashed lines in figure 5*a*.

To quantify if the turning responses differed between fish from high or low predation populations, we first calculated the proportion of times a fish turned towards its partner, out of all its possible turns (in the top two sections of figure 5*a*). While females were more likely than males to make turns towards their partner (*p*_MCMC_ = 0.02), fish from high or low predation populations did not differ in the likelihood of turning towards their partner (*p*_MCMC_ = 0.50). There was also no difference in the mean size of a fish's turn towards its partner between predation regimes (*χ*^2^ = 1.18, d.f. = 1, *p* = 0.28).

Turns can also be used to align with a neighbour's heading, acting to increase polarization between the pair. Alignment responses have seldom been demonstrated in shoaling fish (but see [47]), as often turning is correlated with the position of a neighbour (as above) and not with the heading of that neighbour [22,25]. In guppies, however, we found evidence that turns are also used to align with their neighbour's heading. We partitioned occasions where a neighbour was located to the left or right of a focal individual, and facing towards or away from that individual (figure 5*b*). Fish would most often turn towards a neighbour if the direction to the neighbour was the same sign as the heading of the neighbour (top right and bottom left sections of figure 5*b*). In these cases, the effects of position of the neighbour and heading of the neighbour cannot be uncoupled. Where the signs of the direction to the neighbour and heading of the neighbour are opposite (top left and bottom right sections of figure 5*b*), however, the average turning response is seen to be approximately zero. This is a consequence of averaging two types of responses: (i) either the focal fish turns towards the direction of the neighbour (attraction response) or (ii) the focal fish turns towards the heading of the neighbour (an alignment response). We identified the proportion of times a fish showed alignment responses in these two regions. Females showed alignment responses with their neighbour in 46% of turns, and males showed alignment responses with their partner in 43% of turns. There was no evidence, however, that predation increased the number of alignment responses in females (*p*_MCMC_ = 0.74; electronic supplementary material, table S8) or in males (*p*_MCMC_ = 0.18; electronic supplementary material, Table S8). Nor was there any evidence that the size of the turn to align with a neighbour's heading was different between fish from high or low predation populations (*χ*^2^ = 1.29, d.f. = 1, *p* = 0.26). This result is consistent with the result that groups of eight fish from high or low predation did not differ in their average polarization (see above).

## 4. Discussion

Our results demonstrate that predation shapes the social interaction rules of individuals in moving animal groups. Consistent with previous coarse-scale analyses [13,48], we found that predation increases the cohesion of fish shoals and further demonstrate that this cohesion results from a reduced likelihood of group departure, thereby stabilizing larger group sizes. Our detailed analysis of individuals' movement decisions has revealed that predation shapes fish's attraction–repulsion dynamic, decreasing the critical distance between individuals when they decide to move apart or come back together. Fish from high predation environments achieve increased cohesion despite having larger acceleration and deceleration than fish from low predation environments. There is no evidence, however, that predation shapes individuals' alignment or turning responses, explaining why shoals from high or low predation environments did not differ in group polarization.

Previous studies have suggested that both alignment and attraction responses could be shaped by predation, making group members more cohesive and coordinated with each other [20,21,49]. It appears in this predatory–prey system, predation has shaped the cohesion but not the directional alignment of individuals. Many of the predators of guppies typically attack in short bursts, striking from ambush locations without sustained chases of attack [50,51]. Belonging to a larger group and being closer together, therefore, is perhaps sufficient in reducing individual risk through dilution and selfish herd effects during relatively brief predator encounters in this system. In addition, larger, more cohesive, but not necessarily more aligned groups, can increase the confusion effect making it more difficult for a predator to isolate prey [38,52,53]. An interesting area of research could be to compare the behaviour of fish from high or low predation populations in the presence or absence of predators, or when exposed to different types of predators (e.g. avian or fish predators). This could help highlight how different rules of interaction are selected for, or indeed if the plasticity of anti-predatory responses differ between populations, when prey are exposed to different levels of predation or different predator tactics.

Fish from high predation environments increased cohesion (relative to fish from low predation environments) by decreasing the critical distances at which they decided to move apart or come back together. It will now be of interest to elucidate the finer neurological mechanisms that are responsible for this distance control. The visual system is likely to be the primary sensory modality that is involved in detecting information about the positions and movements of neighbours before a motor decision is initiated. It is interesting to note that the bearing angles at which guppies attempt to position their neighbours (electronic supplementary material, figure S8) are consistent with the theoretical angles that maximize the visual sensitivity for detecting looming objects (such as a neighbour getting closer) and for heading changes of those neighbours [54]. This is consistent with the positioning behaviour of other fish species with stop–start movement [55]. New techniques that detect the sensitivity of retinal cells to approaching and receding objects [56], as well as detailed information on how neighbours are perceived in moving animal groups [29,30] will prove useful in determining whether the sensitivity, or response, to such visual stimuli differs between fish from high or low predation populations.

Another way for individuals in groups to decrease risk is to have effective information transfer between group members [57,58]. Swain *et al.* [55] proposed that the oscillatory movements of fish in schools, like in our study, enriches social information exchange between individuals by breaking the number of occlusions that occur between neighbours [55]. The result that fish from high predation populations were closer together, and performed more, albeit not statistically significant, switches in position than fish from low predation populations is consistent with these interpretations. Predation is likely to shape multiple facets of an individuals' anti-predatory behaviours including group cohesion, but also the propensity for information exchange. This, in turn, may impact how groups make collective decisions together [59].

Fish from high predation environments had larger changes in speed than fish from low predation environments. In males, this difference persisted even when the fish were separated by more than 200 mm, suggesting these responses may not be tailored around social interactions. Indeed, guppies from high predation environments also show stronger acceleration during escape responses compared with fish from low predation environments [44]. Motion creates blur on an animal's retina [60,61] and because of this, detecting moving objects is more difficult with changing speed [62]. Because these fish move with intermittent bursts, it may be more important for fish from high predation environments to minimize the time when excessive motion blur occurs compared with fish from low predation environments. Strengthening both acceleration and deceleration responses could allow for this. Larger acceleration and deceleration responses, however, are likely to be more energetically costly [40], and this may explain why these rapid movements are not adopted across environmental contexts.

In our study, we used wild-caught fish, and therefore cannot disentangle the effects of selection by predation and environmental effects, for example, early life exposure to predators. In Seghers' previous work [13], *F*_3_–*F*_4_ generation fish bred from wild-caught individuals and raised under identical conditions indicated that differences in the schooling behaviour between populations were heritable. It seems likely, therefore, that the effects we observed would also be heritable, although future studies will need to confirm this. Nevertheless, by comparing the collective movement of fish from high and low predation populations, we have provided strong evidence that predation shapes the interaction rules of shoaling fish. Our method to detect the discrete movement decisions made by individuals in moving animal groups also provides a technique to analyse how animals with intermittent forms of locomotion move together. A combination of these analytical techniques, combined with comparative studies and detailed models of collective motion [63–65], will lead to an integrated understanding of how the interaction rules that drive collective motion have been shaped by natural selection.

## Supplementary Material

Supplementary Information

## Data accessibility

All data accompanying this manuscript can be downloaded from the following link: https://figshare.com/s/accc597a873aa0567ade.
