# Supplementary material for: How predation shapes the social interaction rules of shoaling fish
Source: Proc Biol Sci. 2017 Aug 30;284(1861):20171126. doi: 10.1098/rspb.2017.1126 (PMC5577484; doi:10.1098/rspb.2017.1126)
Supplement: Supplementary Information [file rspb20171126supp1.pdf]

Supplementary material for: How predation shapes the social interaction rules of shoaling fish

James E. Herbert-Read[1,2,*], Emil Rosén [2], Alex Szorkovszky[2], Christos C. Ioannou[3], Björn Rogell[1], Andrea Perna[4], Indar W. Ramnarine[5], Alexander Kotrschal[1], Niclas Kolm[1], Jens Krause[6,7], David J.T. Sumpter[1]

[1] Department of Zoology, Stockholm University, 10691, Stockholm, Sweden, [2] Department of Mathematics, Uppsala University, 75106, Uppsala, Sweden, [3] School of Biological Sciences, University of Bristol, Bristol, UK, [4] Department of Life Sciences, Roehampton University, London, UK, [5] Department of Life Sciences,The University of The West Indies, St. Augustine, Trinidad and Tobago, [6] Faculty of Life Sciences, Albrecht Daniel Thaer-Institut, Humboldt-University zu Berlin, Invalidenstrasse 42, 10115 Berlin, Germany [7] Leibniz-Institute of Freshwater Ecology and Inland Fisheries, Department of Biology and Ecology of Fishes, Müggelseedamm 310, 12587 Berlin, Germany ∗ E-mail: Corresponding author james.herbert.read@gmail.com

1 Supplementary Methods and Analyses

1.1 Trials with eight fish

1.1.1 Preprocessing of tracks

All analyses were performed using MATLAB (2016a) and we made use of the inbuilt MATLAB functions where noted. Track segments generated from the Didson tracking software were stitched together using a custom built script. These were then smoothed using a Savitzky-Golay filter with span 7, and degree 4 using the intrinsic *smooth* function. The coordinates of each fish were located in a cartesian coordinate system, with each fish i at time t represented as $(x_i(t), y_i(t))$. These coordinates, measured in pixels, were then transformed into coordinates in mm, calibrated using a known length within the arena.

1.1.2 Distance to centroid

On each frame, we calculated the global centroid as the mean coordinates of all fish in the arena; $(\bar{x}(t), \bar{y}(t))$. The mean distance between each fish's coordinate and the global centroid was then calculated and averaged across trials (Fig. 1).

1.1.3 Detection of subgroups

After lifting the holding cylinder, the fish took approximately one minute before they began to explore the arena. This is observed in Figure 1, where the first minute of the trial involves fish emerging from the holding cylinder and hence the mean distance to the centroid is typically low (because not all fish have emerged). Therefore, in the following analyses, we discarded trajectories within the first minute from when the first fish had been detected as emerging.

To identify the fish that were exploring the arena together in a 'subgroup', for each fish in turn, and on each frame, we identified all neighbours that were within 100 mm of the focal fish. We chose 100 mm because 4-5 body lengths is commonly used to determine group membership in fish shoals [1–3], and the fish were on average 20.5 mm ± 4.3 SD mm length. If a neighbour was within 100 mm of the focal fish, we defined these two fish as 'connected'. We detected all group members that were connected with one another, and also determined the largest one of these connected components using the networkComponents function by Daniel Larremore (see: `http://se.mathworks.com/matlabcentral/fileexchange/42040-find-network-components`). This

component represented the subgroup that contained the largest number of fish that were exploring the arena together on that frame. If there were two subgroups of equally large size on the same frame, then we randomly chose one of the subgroups. We determined the total number of times the largest subgroup size was a group of 8 fish, versus the times when the largest subgroup size contained less than 8 fish across each trial.

1.1.4 Calculation of subgroup size transitional probabilities

For each trial, we determined the modal size of the largest subgroup exploring the arena within 2 second windows (48 frames). We then asked whether this largest subgroup changed in size between successive windows. We obtained, therefore, the total number of times the largest subgroup (of a certain size) increased in size, the total number of times it decreased in size, the total number of times it remained the same size, and hence the total number of possible transitions.

1.1.5 Calculations of the structure and movements of subgroups

The corners of the arenas disrupted the shoaling behaviour of the guppies, often causing them to cross and reducing their directional alignment. To ensure we only analysed times when the fish were shoaling, for the following measures we removed tracks that were within 150 ($\sim$ 130 mm) pixels of the corners of the arena.

To determine the average width and height of the subgroups that were exploring the arena together, on each frame, we rotated the coordinates of fish belonging to a particular subgroup size by the average direction those subgroup members were facing. In effect, the subgroups were rotated so that the group's mean heading was aligned and facing along the positive X axis. The fish at the very front of the group, therefore, had the largest x coordinate and the fish at the back of the group having the smallest x coordinate. We determined the distance between the fish with the smallest and largest x coordinate as a measure of the subgroup's length. In a similar way, the determined the distance between the fish with the largest and smallest y coordinate, giving the subgroups's width at that frame. For each trial, we calculated the mean length and width for each subgroup size.

To visualise the shape of the subgroups of 8 fish as shown in Figure 3 and Figure S2, we produced heat-maps of the positions of group members relative the subgroup's centroid. To construct these heat-maps, we subtracted the centroid from the subgroup's rotated coordinates. This transformed the coordinates so that the group's centroid was located at (0,0) and was facing along the positive X axis. We then determined the number of coordinates that fell within the range x = [-150:1:150] and y = [-150:1:150]. In effect, this represented a 301 x 301 matrix where, the values in each cell of the matrix corresponded to the number of observations that a fish was observed in that position relative to the shoal's centroid. We then summed these matrices from each trial (separately for male or females, and for fish from high or low predation environments). These summed matrices were then normalised by dividing each cell by the maximum number of observations that occurred within one cell of the resultant matrix. For plotting purposes, the resultant heat-maps were rotated anticlockwise by 90 degrees (so that shoals faced along the positive Y axis), and were smoothed with a gaussian filter (using the intrinsic *imgaussfilt* function with sigma = 7). Note that all statistics were performed on the raw data of the subgroup lengths and width, and these plots were purely for visualisation purposes.

To calculate the modal nearest neighbour distances between fish in a trial, we first calculated the distance between each fish and its nearest neighbour on every frame. We then determined the modal nearest neighbour distance between fish in a trial by counting the number of times fish were observed within 0 - 2 mm, 2 - 4 mm, 4 - 6 mm, etc from each other. We then determined the bin with the largest number of observations and used the mean of the bin's edges as a measure of the modal nearest neighbour distance between fish.

On each frame, we also calculated each detected subgroup's centre as the mean coordinates of all fish belonging to that subgroup; $(\bar{x}_{sg}(t), \bar{y}_{sg}(t))$. We then estimated the velocity of each subgroup's centre at time t using:

$$u_c(t) = \frac{\bar{x}_{sg}(t + \Delta t) - \bar{x}_{sg}(t)}{\Delta t} \quad \text{and} \quad v_c(t) = \frac{\bar{y}_{sg}(t + \Delta t) - \bar{y}_{sg}(t)}{\Delta t}. \tag{S1}$$

where Δt is 1/24. We then calculated the speed of the subgroup's centroid as:

$$s_c(t) = \sqrt{(u_c(t))^2 + (v_c(t))^2}. \tag{S2}$$

For each trial, we calculated the median speed of each subgroup size within the trial. We also calculated the median speed of all individuals in each subgroup size.

Polarisation was calculated as $P = \frac{1}{N} \; |\sum_{i=1}^{N} \mathbf{u}_i|$, where $\mathbf{u}_i$ is the unit vector of fish *i*, and *N* are the number of fish in the subgroup. Polarisation scores take values between 0 (no alignment) to 1 (complete alignment). For each trial, we calculated the median polarisation of each subgroup size. We also counted the total number of times subgroups were observed with polarisation scores above 0.85 and the total number of times they were observed. To create the heat plots shown in Figure S5, we determined the number of times a subgroup was observed having speed in the range [0:10:200] mm/s and polarisation scores from [0:0.05:1]. We therefore created a 21 x 21 cell matrix (for each predation and sex combination), where the number in each cell represented the number of times a subgroup had been observed at the speed and polarisation associated with that cell. These maps were normalised by dividing each cell by the sum of all cells. For plotting purposes, these heat-maps were smoothed using a gaussian filter (sigma = 1). As before, however, all statistics were performed on the raw data.

1.2 Trials with pairs of fish

1.2.1 Preprocessing of tracks

Track segments generated from the CTrax were stitched together using a custom built script. Like with the eights, the corners of the arena disrupted the shoaling behaviour of the pairs. Because of this, we did not manually correct tracks that were within 150 ($\sim$ 130 mm) pixels of the corners of the arena, and hence tracks were discarded within these regions. We wanted to ensure that each trial contributed a sufficient amount data in order to accurately quantify the fish's social interactions. Therefore, we only analysed videos where we had at least 2 minutes worth of data for when the pair were shoaling together. Shoaling was defined as when the two fish were less than 100 mm apart (4-5 body lengths) and the fish had moved at least 20 mm within a second. This classification ensured we did not include trials where either the fish had not explored the arena, were not moving, or were not exhibiting shoaling behaviour. Again, we chose 100 mm because this represented 4-5 body lengths of the fish. In practise, the majority of social interactions occurred within this region (Fig. S7A). In total, we removed 30 of the 254 trials (11 from low predation populations and 19 from high predation populations) where data did not reach these criteria.

1.3 Detection of movement decisions

1.3.1 Acceleration and Deceleration Decisions

We determined the x and y components of each fish's, velocity using the standard forward-difference approximations:

$$u_i(t) = \frac{x_i(t+\Delta t) - x_i(t)}{\Delta t} \qquad \text{and} \qquad v_i(t) = \frac{y_i(t+\Delta t) - y_i(t)}{\Delta t}, \tag{S3}$$

where $\Delta t = 1/24$ s was the constant duration between consecutive video frames. A fish's speed at time t was then approximated as:

$$s_i(t) = \sqrt{(u_i(t))^2 + (v_i(t))^2}. \tag{S4}$$

To detect the times when a fish decided to move (increase in speed), we smoothed these speeds using a Savitzky-Golay filter with a span of 12 frames (1/2 second) and degree 3. We then detected the times and heights of the peaks and troughs of these speeds using the *findpeaks* inbuilt function in MATLAB, with a minimum distance of 8 frames (1/3) second between adjacent troughs or peaks (Fig. 4A). Because fish

showed strong directional alignment when they were less than 100 mm apart (Fig. S7A), and because we were interested in how fish updated their position as a function of its neighbour's position when they were shoaling, we only analysed decisions when fish were less than 100 mm apart. Using a custom built script, we paired each trough (magenta points in Fig. 4A) with the following peak (yellow points in Fig. 4A) of the speed profile. Each paired trough and peak was classified as a *decision*. We removed spurious decisions where the change in speed between the trough and peak of a decision was less than 20 mm/s. We determined this cutoff by plotting a histogram of all decision speeds across all trials, which revealed a strong peak below 20 mm/s (Fig. S7B), revealing the spurious decisions (noise). An example of one of these spurious decisions can be seen in Figure 4A at $\sim$ 9.5 seconds. We calculated the acceleration within each of these decisions as the change in speed between the trough and the peak of a decision, divided by the time lag between the trough and the peak of the decision. To calculate the deceleration after a decision, we calculated the change in speed between the decision's peak and the next decision's trough, divided by the time between the decision's peak and the next decision's trough. Because the fish did not always make a second decision immediately after the first decision, we only included decelerations when the time between the decision peak and next decision's trough was less that 15 frames (0.62 seconds). This ensured we captured the true deceleration of the fish, without including long spurious periods of time between non-sequential decisions.

We calculated the distance a fish moved during each of its decisions (Fig. S11A). To do this, we determined the fish's position at the start of its decision (when the fish had its minimum speed) and the fish's position at the start of its next decision. We then calculated the distance between these two points. We only used consecutive decisions if there was less than 50 frames ($\sim$ 2 seconds between these two decisions. This was determined by plotting the probability distribution of the time between decisions (Fig. S11B).

We identified the distance between the pair when a fish made a decision, and determined the acceleration of the fish as a function of this distance (Fig. S10). This indicated that beyond 200 mm, the fish's acceleration were relatively consistent, albeit with a slight decay with distance. We tested whether fish from high or low predation differed in their acceleration when fish were more separated, we calculated the mean acceleration of the fish when fish were separated by more than 200 mm. Because the fish were generally shoaling together, there was far less data in this region than when fish were less than 200 mm apart. We also had to remove clear two outliers from these data for statistical analysis.

To generate the heat-maps shown in Figure 4D-G and Figure S9C-F, for each decision, we determined the relative location of the neighbour with respect to the focal fish that made the decision. Negative and positive x values of the neighbour indicated that the neighbour was to the left or to the right of the focal fish, respectively. Negative and positive y values of the neighbour indicated that the neighbour was behind or in front of the focal fish, respectively. We then discretised these locations in the range [-100:5:100] mm in both the x axis and the y axis. We then determined the mean acceleration and deceleration of fish when their neighbour was in each of these discretised locations across all decisions, but separately for males or females, and for fish from high or low predation populations. For plotting purposes, these heat-maps were smoothed with a Gaussian filter, with sigma = 4. We also determined the total number of decisions that were made when neighbours were in these locations, which was used to determine the probability contour regions using the *probcontour* function. Note that the statistics were performed on the raw measures, and these plots were purely for visualisation purposes.

At each decision point, we identified the fish that was in front or behind the other fish relative to the pair's direction of travel. To remove any ambiguity about which fish was in front or behind the other, we only assessed this positioning when the pair's polarisation was above 0.5. We then determined how the distance between the pair varied in the two seconds following a decision by either the lead fish or the following fish (see Fig. 4A). We also determined whether the pair switched positions during these decisions, by assessing whether the fish that was at the front of the pair remained at the front of the pair 15 frames (0.62 seconds) after either fish had made a decision. If the two fish had changed position, we counted this as a switch. The number of times the fish did not switch position during these decisions was also counted.

To determine a decision rate for each fish, we counted the number of decisions a fish made, and divided

this by the number of seconds the fish had been observed less than 100 mm from its partner. On average, males updated their position at a rate of 0.84 movements s^{-1} ($\pm$ 0.02 SE) whereas the larger females updated their position at a rate of 0.65 movements s^{-1} ($\pm$ 0.02 SE). Pairs of fish did not update their position at the same time. For each fish, we determined the time delay between when one fish moved, and then the next fish moved. To ensure the decision made by the first fish could have been influencing the decision made by the second fish, we set a 'cut-off' time for when the second fish had to have made a decision after the first fish's decision, if these decisions were to be counted as paired. Because males made on average 0.84 decisions per second, this cut-off time was set to 28.6 frames (1.19 s), and because females made on average 0.65 decisions per second, this cut-off was set to 37.1 frames (1.54 s). Males updated their position 0.47 (± 0.003 SD) seconds after their neighbour had moved, whereas females updated their position 0.54 (± 0.004 SD) seconds after their neighbour had moved.

1.3.2 Turning Decisions

Each fish's heading was calculated from the ellipse fitted to each fish's body orientation during tracking [4] and took values between 0 and 2π. These headings were made continuous by unwrapping heading changes around 2π. For example, we converted a heading change from 350 to 10 degrees to 350 to 370 degrees. This removed any discontinuity around 0 and 2π allowing us to compare changes in angles around this junction. This also assumes that fish do not turn more than 180 degrees in one frame, and because the fish are not exhibiting escape responses, our temporal resolution (24 fps) should have removed this possibility. The fish typically travelled at the same heading, interspersed with sharp changes in heading (Fig. S7C). To detect these changes in heading, we used an algorithm developed by Weinmann *et al.* (2015) [5]. The function *minL1Potts.m* was applied to the unwrapped headings of a fish with $\gamma = 5$. This produced a stepwise function highlighting the changes in direction (Fig. S7C). Since the algorithm marks the step-points in the middle of the change in angle, and not when the final heading is reached, we deducted 6 frames (0.25 seconds) from the timestamps of the direction changes to determine the instance when a fish started to change its heading. All code associated with these analyses can be downloaded from `http://pottslab.de/`. The size and direction of a turn, therefore, was calculated as the change in angle between successive changes in heading. Positive turn angles indicate the fish turned to the left, whilst negative turn angles indicate the fish made a turn to the right. We also assessed the rate at which fish made turns by calculating the number of turns performed, divided by the total time they were less than 100 mm apart. Predation did not affect the turning rates of males or females (Table S5), however, smaller females made more turns than larger females (χ^2= 4.97, d.f = 1, *P* = 0.03).

To create the heat-map in Figure 5A, like the acceleration heat-maps, we determined the relative location of the neighbour when a fish made a turning decision. Again, these relative locations were discretised in the range [-100:5:100] mm in both the x and the y axes. We then determined the average turn angle when neighbours were in each of these discretised locations. This is represented by the colour in Figure 5A. For plotting purposes, these heat-maps were smoothed with a Gaussian filter, sigma = 2. These heat-maps revealed a fish's turning responses could be broken down into 3 distinct regions as a function of their neighbour's position. To define these regions, we divided the relative location of the neighbour into three 120 degree sections, indicated by the dotted lines in Figure 5A. In each trial, we counted the total number of turns a fish made when their neighbour was in each of these top two sections. We also counted the number of times a fish turned left or right when their neighbour was in each of these top two sections. In each trial, therefore, we determined the number of times a fish turned towards its neighbour out of the total number of turns it made. We also determined the mean size of the turns that were directed towards the location of the neighbour in these top two sections within each trial.

To create the heat-map in Figure 5B, for each turning decision, we determined the relative direction of the neighbour with respect to the focal fish, and the heading of the neighbour. These directions and heading were discretised in the range $[-\pi : 2\pi/49 : \pi]$. With regards to the direction of the neighbour, negative values of π indicate a neighbour was to the right of the focal individual making the turn, and positive values of π indicate

a neighbour was to the left of the focal individual making the turn. We determined the mean turn size when neighbours were within each of these discretised direction-heading combinations. This is shown as the colour in Figure 5B, smoothed with a Gaussian filter, sigma = 2. Where the signs of the direction and heading of the neighbour are the same, the neighbour is located to the left or right of the focal individual and facing away from it. In these cases, turns towards a neighbour could either be due to attraction responses (turns towards position of the neighbour), alignment responses (turns to align with the direction of the neighbour) or both responses combined. Note that if the fish only performed turns towards their neighbour's position, then this heat-map could be broken down into just two regions along the line X = 0. This is not the case, and the turning responses as a function of the heading and direction of a fish's neighbour, need to be broken down into four regions as indicated by the dotted lines in Figure 5B. In the two regions where the signs of the direction and heading of the neighbour are opposite, we counted the number of times a fish turned to align with their neighbour's heading, out of the total number of turning decisions in these regions. This was determined separately for each trial. We also determine the mean size of the turn that would have resulted in the fish becoming aligned within these two regions in each trial.

1.4 Statistics

1.4.1 General Model Formulation

We modelled all response variables using generalised linear mixed effects models. These were fitted with predation regime (high or low), sex, subgroup size (where applicable) and body size (see Fig. S1) as fixed effects. River (nested within predation and crossed with sex), and trial (where applicable) were fitted as random factors to the data. Sex, Predation and subgroup size (where applicable) were treated as categorical variables in all analyses, whereas body size was treated as a continuous. We assessed the interactions of predation and sex, but not subgroups size (where applicable) or body size, because these are expected to scale with our shoaling measures. The full fixed effect models were simplified by removal of non-significant terms. If interactions were observed between predation and sex, without an overall significant predation effect, we analysed sexes in separate models. In practice, we found significant interactions between predation and sex in the polarisation of groups of eight fish ($\chi^2 = 4.81$, df = 1, $P = 0.028$), the deceleration of pairs ($\chi^2 = 6.19$ df = 1,$P = 0.013$), the proportion of alignment responses in pairs (pMCMC = 0.048), the mean response lag time ($\chi^2 = 6.88$ df = 1,$P = 0.009$), and the decision rates (speed and turning rates) in pairs (speed: $\chi^2 = 13.9$ df = 1,$P < 0.001$; turning: $\chi^2 = 4.69$ df = 1,$P < 0.03$). All statistical analyses were performed in R.

1.4.2 Binomially Distributed Variables

Binomially distributed variables (i.e. increase/decrease of shoal size, observations in/out of a high polarised state, number of switches/non switches) were typically over-dispersed, and therefore all binomially distributed data were modelled with Markov-Chain Monte Carlo Generalised Linear Mixed Models (MCMCglmm) that allows for over dispersion. All MCMCglmm models were run for 4040000 iterations after a burnin of 40000 and a thinning interval of 4000 to yielding a total posterior sample of 1000 posterior samples per chain. Flat priors were used for the fixed effects and locally uninformative priors were used for the random effects, both representing little prior knowledge. Convergences of each model were assessed using diagnostic plots (using the scapeMCMC library in *R*) and using the Gelman-Rubin diagnostic statistic [6] implemented through the coda package in R [7]. We ran duplicates of all models with over-dispersed starting values, and then compared the mixing properties of chains between identical models. All models showed adequate convergence (multivariate potential scale reduction factors all <1.02). All auto-correlations were within the interval -0.1 and 0.1.

1.4.3 Continuous Variables

Continuous response variables were modelled with linear mixed effects models (LME). These models were fitted using maximum likelihood. To avoid violating LME assumptions (i.e. residual distribution and hetero-

scedasticity which were checked with diagnostic plots), shoal width, shoal length, modal distance (eights), average distance moved during acceleration/deceleration periods, mean alignment response during turns, and mean decision lag were log transformed. The polarisation of groups was -log(1-P) transformation, where P was the median polarisation of the subgroup. All other variables were untransformed.

2 Supporting Figures

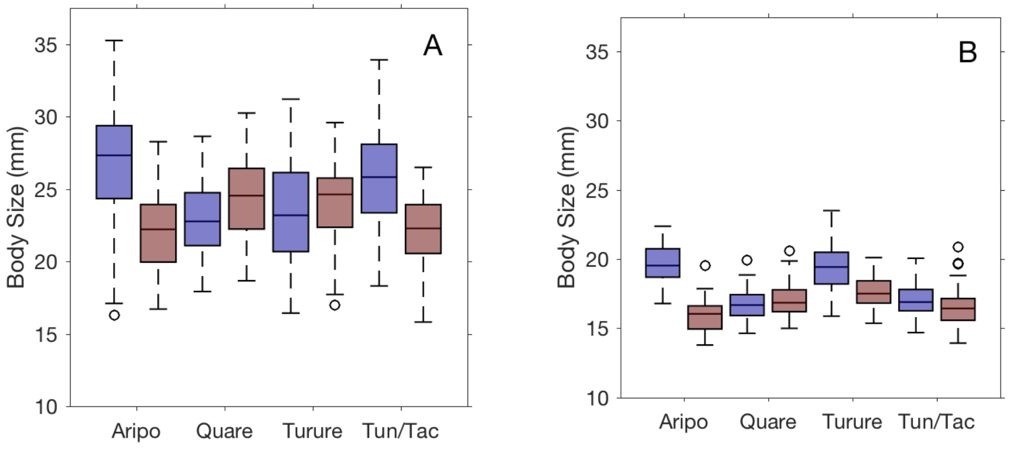

Figure S1. Mean (± 1 SD) size of females (A) or males (B) from the different populations. Tun/Tac is the Tunapuna and Tacarigua river. Note that these plots show body sizes for all fish used in pairs and in groups of 8 combined. The horizontal line in the centre of each box denotes the median of each category, whilst the bottom and top edges of each box denote the 25th and 75th percentiles, respectively. The whiskers of each boxplot extends to the data points that are not considered outliers.

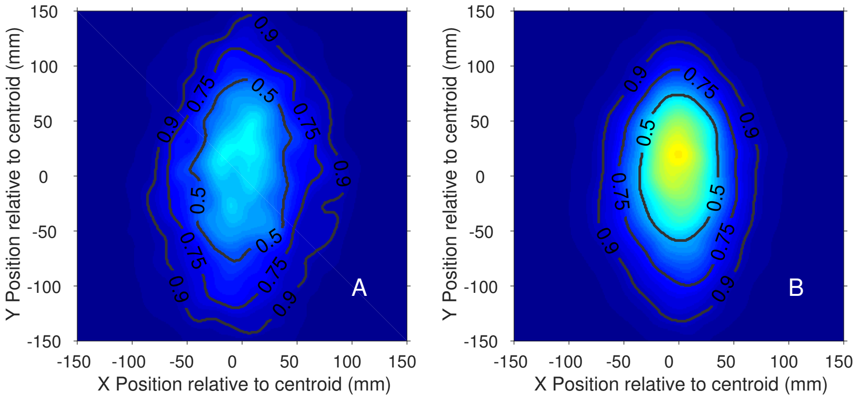

Figure S2. Shape of shoals of 8 male fish from low predation (A) or high predation (B) populations. Contour lines represent regions containing the proportion of total observations where individuals were found relative to the shoal centroid located at (0,0). Shoals from high predation populations were generally more compact than shoals from low predation populations. These patterns were consistent in shoals of 8 female fish (Fig. 3) and across different subgroup sizes (Fig. S3).

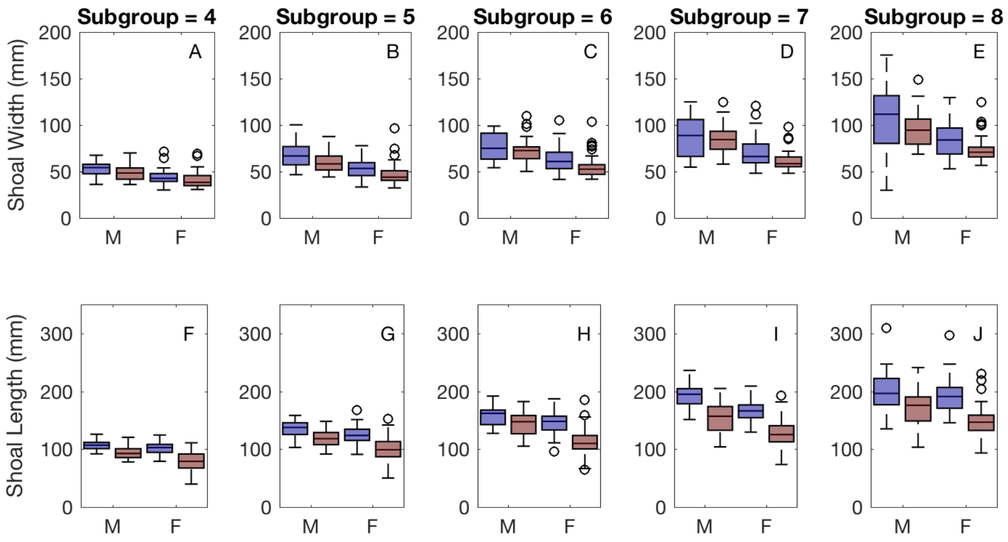

Figure S3. Boxplots of the width and length of shoals after separating them according to their subgroup size (categorised when individuals were within 100 mm of each other). Males and females from high predation populations (red bars) have smaller shoal widths and lengths than fish from low predation populations (blue bars). The horizontal line in the centre of each box denotes the median of each category, whilst the bottom and top edges of each box denote the 25th and 75th percentiles, respectively. The whiskers of each boxplot extends to the data points that are not considered outliers.

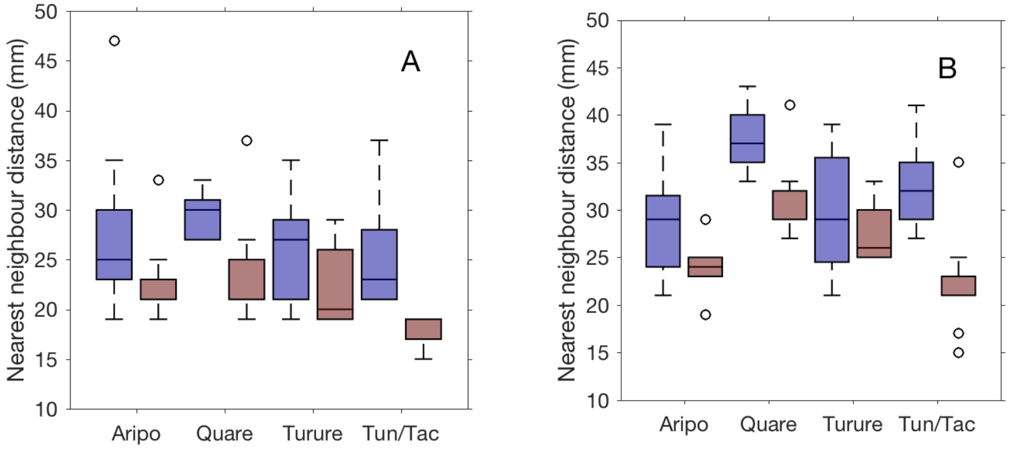

Figure S4. Modal nearest neighbour distances for groups of eight (A) female or (B) male fish from high predation (red) or low predation (blue) populations. The horizontal line in the centre of each box denotes the median of each category, whilst the bottom and top edges of each box denote the 25th and 75th percentiles, respectively. The whiskers of each boxplot extends to the data points that are not considered outliers. Fish from high predation populations had smaller modal nearest neighbour distances than fish from low predation populations (χ^2= 17.0, d.f. $= 1$, $P < 0.001$), and females had smaller modal nearest neighbour distances than males (χ^2= 10.6, d.f. $= 1$, $P < 0.01$)

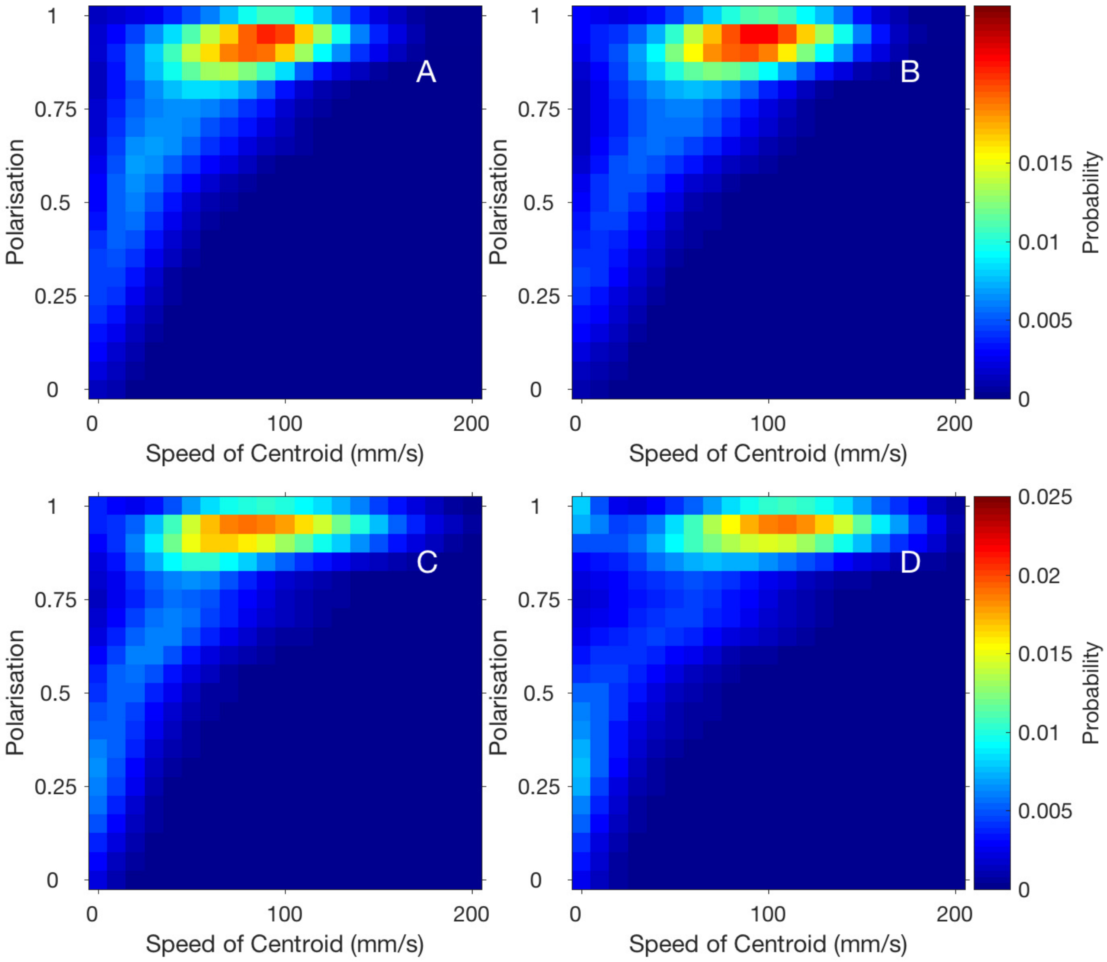

Figure S5. Speed of the shoal centroid versus shoal polarisation for 8 fish. Faster shoals were generally more polarised, although the polarisation of groups was unaffected by predation regime. The heat shows the probability of finding groups in each binned region of the parameter space. (A & B) Males from (A) low or (B) high predation populations. (C&D) Females from (C) low or (D) high predation populations.

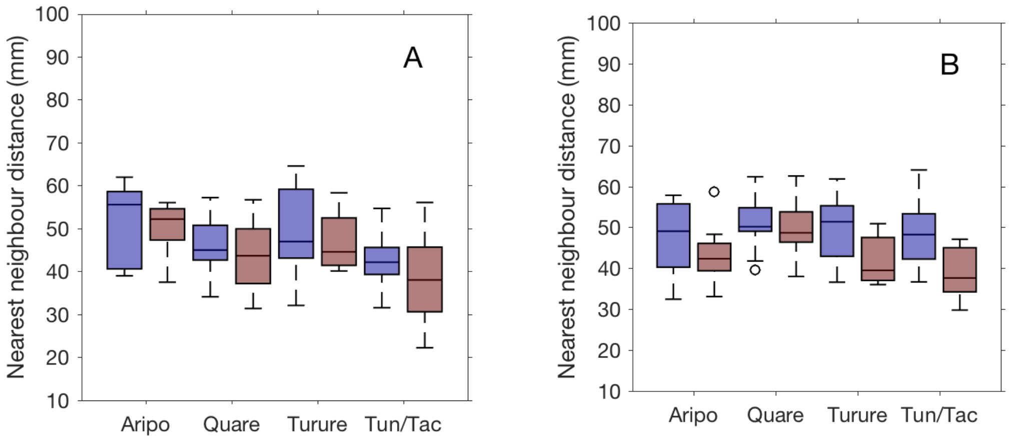

Figure S6. Mean (± 1 SE) median neighbour distance for (A) female and (B) male pairs. Low (blue) and high (red) predation populations of the same river are shown side by side for comparison. Tun/Tac is the Tunapuna and Tacarigua river. Note we use median distances here because modal neighbour distances for pairs are not reliable due to insufficient data required for accurate binning. The horizontal line in the centre of each box denotes the median of each category, whilst the bottom and top edges of each box denote the 25th and 75th percentiles, respectively. The whiskers of each boxplot extends to the data points that are not considered outliers.

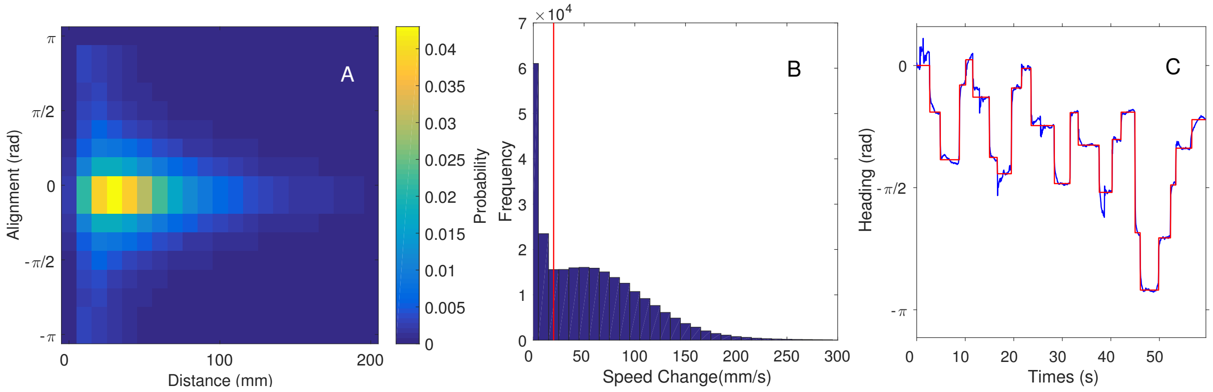

Figure S7. (A) We filtered the movement decisions so that we only analysed decisions when fish were less than 100 mm apart. The majority of interactions occur within this region. (B) Histogram of all changes in speed during decisions (change in height of paired magenta points to yellow points in Fig. 4A). We filtered decisions that were less than 20 mm/s changes in speed (red line), as these represented spurious decisions likely due to noise (i.e. decision at approximately 9.5 s in Fig. 4A). (C) Example of a guppy's turning profile. Guppies swim in the same direction with intermittent changes in heading. We used a step detection algorithm to detect these changes in direction. Blue line represents the original headings calculated during tracking. The red line represents the step changes detected. The times of these changes are plotted in Fig. 4A as the dashed lines.

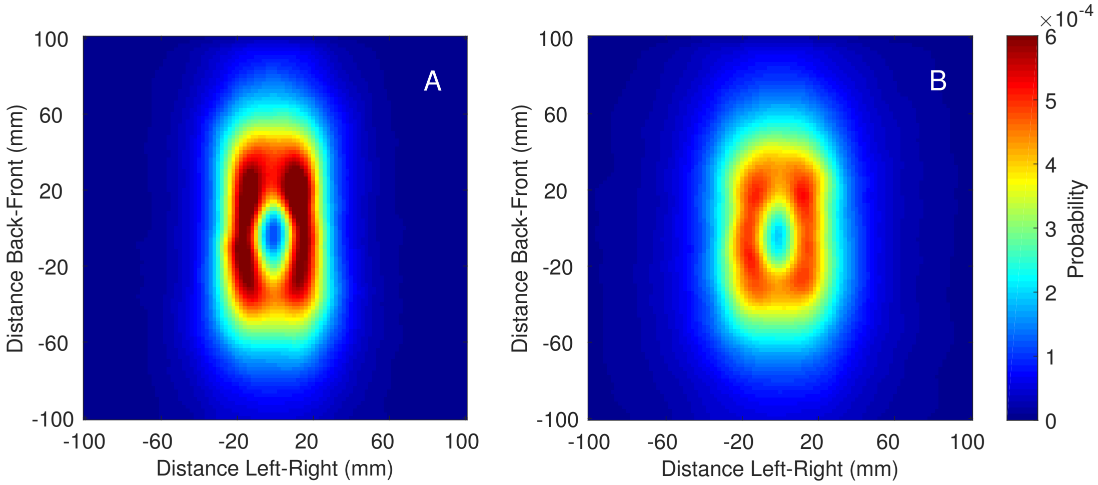

Figure S8. Probability of finding a neighbour in different locations around a focal fish locating in the centre of the plot. (A) Female pairs (B) Male pairs. Fish are generally observed ∼ 15 - 20 mm apart side-by-side, with more varying distances apart in their direction of motion. For plotting purposes, the heatplots were blurred using a guassian filter, sigma = 2.

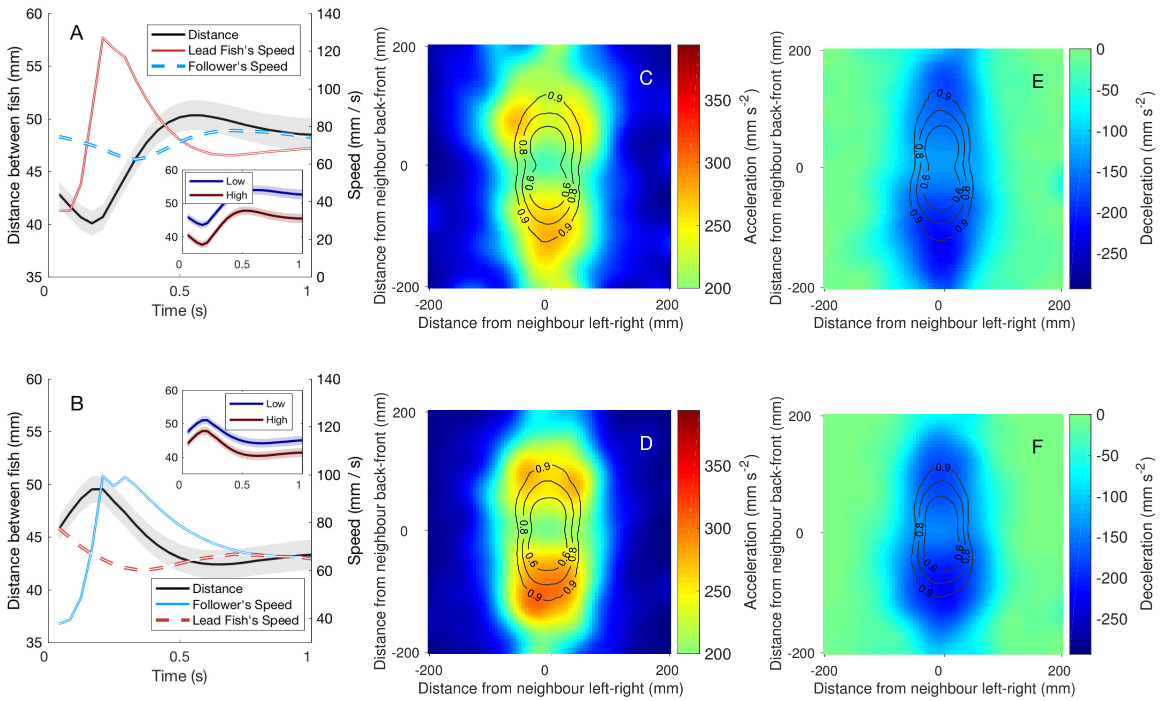

Figure S9. How pairs of female guppies maintain their separation distances. (A) Speed profile of the fish at the front of the pair (red line - refer to right Y axis) when it decides to move (at 0 seconds). The lead fish decides to move when the distance between the fish reaches $\sim$ 43 mm (averaged over both high and low predation females), but these distances are significantly lower in fish from high predation populations (red line in insert) than fish from low predation populations (blue line in insert). (B) Speed profile of the follower (light blue line - refer to right Y axis) when it decides to move. The follower speeds up when the distance between the fish reaches $\sim$ 46 mm (averaged over both high and low predation females), but again, these distances are significantly lower in fish from high predation populations (red line in insert) than fish from low predation populations (blue line in insert). Error bars in (A) & (B) represent mean standard error per trial (only partially visible for speed due to low variation). Data are only for female fish, but males also show the same movement profile, with similar separation distances (Fig. 4) (C&D) Acceleration of female fish from low (C) or high (D) predation populations as a function of neighbour position. (E&F) Deceleration of female fish from low (E) or high (F) predation populations as a function of neighbour position. Females from high populations have higher acceleration than their low predation counterparts. Deceleration in females is related to body size, with smaller fish having larger decelerations. Data from lead fish and followers are combined within these plots since they show similar symmetry around y = 0.

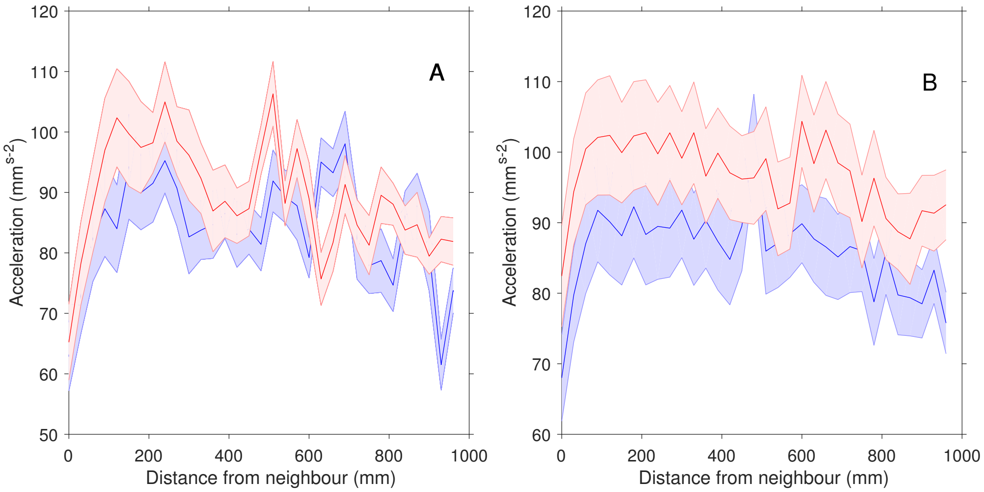

Figure S10. Mean acceleration (± 1 SE) of fish during their movement decisions as a function of their distance from their partner. (A) females or (B) males from low (blue) or high (red) predation environments.

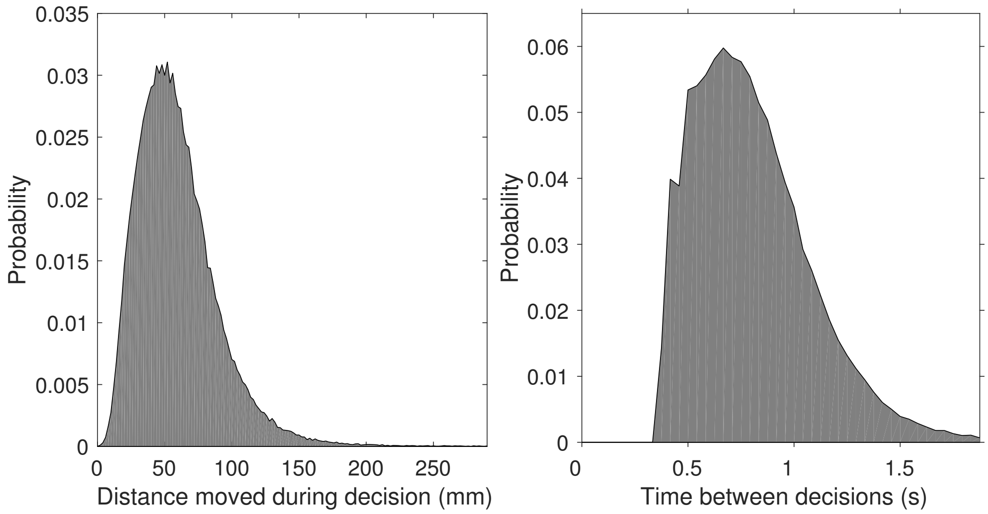

Figure S11. (A) Probability of the distance a fish moved during a decision (B) Probability of the time between consecutive decisions.

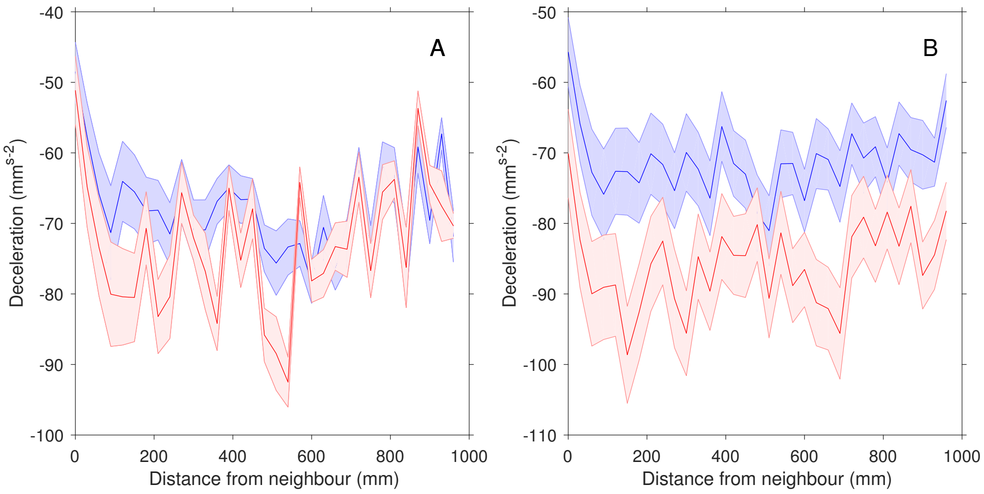

Figure S12. Mean Deceleration ($\pm$ 1 SE) of fish during their movement decisions as a function of their distance from their partner. (A) females or (B) males from low (blue) or high (red) predation environments.

3 Supporting Tables

Table S1. Bayesian GLMM of the proportion of time fish were observed in a group of 8

	Mean	95% CI	pMCMC
**Fixed Effects**			
(Intercept)	-1.09	-2.39 to 0.28	0.096
Predation (+)	1.14	0.32 to 1.93	0.012
Sex (M)	-2.31	-3.37 to -1.23	0.006
Body size	0.25	-0.19 to 0.62	0.234
**Random Effects**			
Predation:River	0.157	$3.0e-08$ to 0.6	
River	1.217	$1.3e-08$ to 3.9	
Sex:Predation:River	0.069	$2.5e-08$ to 0.3	
River:Sex	0.242	$2.7e-07$ to 1.0	
Trial	1.081	$4.4e-06$ to 1.8	

Table S2. Bayesian GLMM of transitions between different subgroup sizes.

(a) Increase Transitions

	Mean	95% CI	pMCMC
**Fixed Effects**			
(Intercept)	-0.3861	-1.330 to 0.6061	0.394
Predation (+)	0.0772	-0.302 to 0.4036	0.588
Sex (M)	-0.4043	-0.781 to -0.0092	0.040
Subgroup (4)	-0.6522	-0.846 to -0.4540	0.001
Subgroup (5)	-0.7726	-0.977 to -0.5638	0.001
Subgroup (6)	-0.9642	-1.161 to -0.7514	0.001
Subgroup (7)	-0.9470	-1.170 to -0.7523	0.001
Body size	-0.0028	-0.034 to 0.0326	0.840
**Random Effects**			
Predation:River	0.0496	$1.7e-07$ to 0.173	
River	0.1940	$7.1e-07$ to 0.653	
Sex:Predation:River	0.0272	$6.8e-08$ to 0.089	
River:Sex	0.0427	$5.3e-08$ to 0.163	
Trial	0.0069	$8.1e-08$ to 0.026	

(b) Decrease Transitions

	Mean	95% CI	pMCMC
**Fixed Effects**			
(Intercept)	-1.869	-3.33 to -0.493	0.004
Predation (+)	-0.699	-1.34 to -0.222	0.026
Sex (M)	1.107	0.56 to 1.716	0.002
Subgroup (5)	0.467	0.30 to 0.623	0.001
Subgroup (6)	0.551	0.38 to 0.727	0.001
Subgroup (7)	0.694	0.52 to 0.860	0.001
Subgroup (8)	0.669	0.52 to 0.849	0.001
Body size	-0.026	-0.07 to 0.026	0.312
**Random Effects**			
Predation:River	0.119	$9.7e-08$ to 0.42	
River	0.309	$6.2e-07$ to 1.23	
Sex:Predation:River	0.055	$6.6e-09$ to 0.18	
River:Sex	0.083	$8.8e-07$ to 0.31	
Trial	0.158	$8.8e-02$ to 0.24	

Table S3. ANOVA outputs of the GLMMs on continuous variables for the shoals of eight fish

(a) Shoal Width (log)

	χ^2	Df	Pr(>χ^2)
Predation	4.86	1	0.0274
Sex	15.62	1	<0.001
Subgroup Size	1993	4	<0.001
Body size	0.17	1	0.68

(b) Shoal Length (log)

	χ^2	Df	Pr(>χ^2)
Predation	10.50	1	<0.01
Sex	17.04	1	<0.001
Subgroup Size	3134.51	4	<0.001
Body size	0.01	1	0.94
Predation*Sex	12.69	1	<0.001

(c) Modal Distance Apart (log)

	χ^2	Df	Pr(>χ^2)
Predation	14.94	1	<0.001
Sex	10.81	1	<0.01
Body size	0.68	1	0.41

(d) Polarisation - Females (-log(1-P))

	χ^2	Df	Pr(>χ^2)
Predation	0.34	1	0.5620
Subgroup Size	152.37	4	0.0000
Body size	0.14	1	0.7106

(e) Polarisation - Males (-log(1-P))

	χ^2	Df	Pr(>χ^2)
Predation	1.09	1	0.2974
Subgroup Size	79.36	4	0.0000
Body size	1.31	1	0.2516

(f) Centroid Speed

	χ^2	Df	Pr(>χ^2)
Predation	0.10	1	0.75
Sex	0.07	1	0.79
Subgroup Size	12.28	4	0.02
Body size	0.11	1	0.74

(g) Individuals' Median Speed

	χ^2	Df	Pr(>χ^2)
Predation	0.16	1	0.69
Sex	0.00	1	0.98
Subgroup Size	6.09	4	0.19
Body size	0.27	1	0.61

Table S4. Bayesian GLMM of the proportion of time a group was observed in a highly polarised state (P > 0.85)

	Mean	95% CI	pMCMC
**Fixed Effects**			
(Intercept)	1.031	0.27 to 1.694	0.018
Predation (+)	0.007	-0.55 to 0.590	1.000
Sex (M)	-0.167	-0.68 to 0.328	0.506
Subgroup (5)	-0.124	-0.23 to -0.024	0.018
Subgroup (6)	-0.122	-0.24 to -0.023	0.026
Subgroup (7)	-0.269	-0.38 to -0.160	0.001
Subgroup (8)	-0.297	-0.41 to -0.188	0.001
Body size	-0.070	-0.21 to 0.096	0.376
**Random Effects**			
Predation:River	0.121	$1.9e-06$ to 0.46	
River	0.299	$3.8e-08$ to 0.91	
Sex:Predation:River	0.096	$1.9e-04$ to 0.25	
River:Sex	0.058	$3.7e-08$ to 0.26	
Trial	0.151	$1.0e-01$ to 0.21	

Table S5. ANOVA outputs of the GLMMs on continuous variables for the shoals of two fish

(a) Median Distance Apart

	χ^2	Df	Pr(>χ^2)
Predation	9.89	1	0.0017
Sex	1.34	1	0.2464
Body size	4.77	1	0.0289

(b) Initiation Distance

	χ^2	Df	Pr(>χ^2)
Predation	7.13	1	0.0076
Sex	2.34	1	0.1262
Body size	4.66	1	0.0309

(c) Mean Acceleration

	χ^2	Df	Pr(>χ^2)
Predation	5.21	1	0.0224
Sex	4.37	1	0.0366
Body size	6.98	1	0.0083

(d) Acceleration females > 200 mm apart

	χ^2	Df	Pr(> χ^2)
Predation	0.10	1	0.7568
Body size	0.05	1	0.8299

(e) Acceleration males > 200 mm apart

	χ^2	Df	Pr(>χ^2)
Predation	7.95	1	0.0048
Body size	1.81	1	0.1789

(f) Distance travelled during deceleration period (log)

	χ^2	Df	Pr(>χ^2)
Predation	0.13	1	0.7200
Sex	0.51	1	0.4738
Body size	0.32	1	0.5710

(g) Mean Deceleration (females)

	χ^2	Df	Pr(>χ^2)
Predation	0.34	1	0.5586
Body size	10.09	1	0.0015

(h) Mean Deceleration (males)

	χ^2	Df	Pr(>χ^2)
Predation	9.73	1	0.0018
Body size	1.38	1	0.2393

(i) Mean Deceleration (females) > 200 mm apart

	χ^2	Df	Pr(>χ^2)
Predation	0.41	1	0.5231
Body size	2.57	1	0.1092

(j) Mean Deceleration (males) > 200 mm apart

	χ^2	Df	Pr(>χ^2)
Predation	6.61	1	0.0102
Body size	0.60	1	0.4389

(k) Mean Turn Size

	χ^2	Df	Pr(>χ^2)
Predation	1.18	1	0.2768
Sex	3.02	1	0.0824
Body size	0.11	1	0.7423

(l) Mean alignment turning angle (log)

	χ^2	Df	Pr(>χ^2)
Predation	1.29	1	0.2561
Sex	0.49	1	0.4843
Body size	0.28	1	0.5979

(m) Mean lag between pairs' decisions (log) - females

	χ^2	Df	Pr(>χ^2)
Predation	0.27	1	0.6021
Body size	1.07	1	0.3001

(n) Mean lag between pairs' decisions (log) - males

	χ^2	Df	Pr(>χ^2)
Predation	1.85	1	0.1734
Body size	2.89	1	0.0890

(o) Mean decision rate - females

	χ^2	Df	Pr(>χ^2)
Predation	7.92	1	0.0049
Body size	0.38	1	0.5392

(p) Mean decision rate - males

	χ^2	Df	Pr(>χ^2)
Predation	0.88	1	0.3491
Body size	4.45	1	0.0349

(q) Mean turning rate - females

	χ^2	Df	Pr(>Chisq)
Predation	0.31	1	0.5753
Body size	4.97	1	0.0257

(r) Mean turning rate - males

	χ^2	Df	Pr(>Chisq)
Predation	0.57	1	0.4514
Body size	0.04	1	0.8492

Table S6. Bayesian GLMM of the number of decisions that resulted in a switch between the lead fish and the follower.

	Mean	95% CI	pMCMC
**Fixed Effects**			
(Intercept)	-2.059	-2.533 to -1.677	0.001
Predation (+)	0.285	-0.034 to 0.682	0.092
Sex (M)	0.135	-0.108 to 0.376	0.246
Body size	-0.084	-0.185 to 0.011	0.106
**Random Effects**			
Predation:River	0.0635	$1.4e-08$ to 0.208	
River	0.0919	$4.2e-08$ to 0.409	
Sex:Predation:River	0.0076	$5.8e-09$ to 0.030	
River:Sex	0.0107	$4.3e-09$ to 0.048	

Table S7. Bayesian GLMM of the proportion of turns made towards the location of a neighbour

	Mean	95% CI	pMCMC
**Fixed Effects**			
(Intercept)	1.485	0.82 to 2.013	0.002
Predation (+)	-0.131	-0.55 to 0.357	0.504
Sex (M)	-0.486	-0.84 to -0.181	0.024
Body size	-0.031	-0.12 to 0.073	0.524
**Random Effects**			
Predation:River	0.1130	$1.0e-08$ to 0.356	
River	0.2176	$7.0e-08$ to 0.874	
Sex:Predation:River	0.0091	$1.1e-07$ to 0.038	
River:Sex	0.0416	$1.6e-07$ to 0.154	

Table S8. Bayesian GLMM of the proportion of alignment responses

(a) Females

	Mean	95% CI	pMCMC
**Fixed Effects**			
(Intercept)	-0.186	-0.556 to 0.18	0.23
Predation (+)	0.044	-0.277 to 0.37	0.74
Body size	-0.014	-0.083 to 0.06	0.72
**Random Effects**			
Predation:River	0.051	$2.1e-05$ to 0.17	
River	0.134	$1.5e-07$ to 0.57	

(b) Males

	Mean	95% CI	pMCMC
**Fixed Effects**			
(Intercept)	-0.202	-0.570 to 0.131	0.18
Predation (+)	-0.191	-0.504 to 0.131	0.18
Body size	-0.019	-0.066 to 0.038	0.49
**Random Effects**			
Predation:River	0.049	$3.0e-04$ to 0.16	
River	0.091	$8.6e-09$ to 0.39	

References

1. Pitcher T, Magurran A, Allan J (1983) Shifts of behaviour with shoal size in cyprinids in *Proceedings of the British Freshwater Fisheries Conference*. Vol. 3, pp. 220–228.

2. Pitcher TJ (1986) Functions of shoaling behaviour in teleosts in *The behaviour of teleost fishes*. (Springer), pp. 294–337.

3. Hoare D, Couzin ID, Godin JG, Krause J (2004) Context-dependent group size choice in fish. *Animal Behaviour* 67(1):155–164.

4. Branson K, Robie AA, Bender J, Perona P, Dickinson MH (2009) High-throughput ethomics in large groups of drosophila. *Nat Methods* 6(6):451–457.

5. Weinmann A, Storath M, Demaret L (2015) The lˆ1-potts functional for robust jump-sparse reconstruction. *SIAM J Numer Anal* 53(1):644–673.

6. Gelman A, Rubin DB (1992) Inference from iterative simulation using multiple sequences. *Stat Sci* pp. 457–472.

7. Plummer M, Best N, Cowles K, Vines K (2006) Coda: Convergence diagnosis and output analysis for mcmc. *R news* 6(1):7–11.
